# A Novel *KDF1* Variant is Associated With Multiple Natal Teeth, Tooth Agenesis, and Root Maldevelopment

**DOI:** 10.1016/j.identj.2025.100860

**Published:** 2025-06-23

**Authors:** John M. Graham, Pedro A. Sanchez-Lara, Atsushi Ohazama, Katsushige Kawasaki, Stefan T. Arold, Piranit Nik Kantaputra

**Affiliations:** a Department of Pediatrics, Division of Medical Genetics, Guerin Children’s Hospital at Cedars-Sinai Medical Center, and David Geffen School of Medicine at UCLA, Los Angeles, California, USA; b Division of Oral Anatomy, Faculty of Dentistry & Graduate School of Medical and Dental Sciences, Niigata University, Niigata, Japan; c KAUST Center of Excellence for Smart Health, Biological and Environmental Science and Engineering Division, King Abdullah University of Science and Technology (KAUST), Thuwal, Saudi Arabia; d Center of Excellence in Medical Genetic Research, Faculty of Dentistry, Chiang Mai University, Chiang Mai, Thailand; e Department of Orthodontics and Pediatric Dentistry, Division of Pediatric Dentistry, Faculty of Dentistry, Chiang Mai University, Chiang Mai, Thailand

**Keywords:** Natal teeth, Multiple natal teeth, Tooth agenesis, Taurodontism, Hypodontia, Oligodontia, Root maldevelopment, Unseparated roots

## Abstract

**Objective:**

Natal teeth are teeth that are present at birth. Multiple natal teeth are extremely rare. The objective of this study was to find the molecular aetiology of a unique dental phenotype including natal teeth, tooth agenesis, and root maldevelopment in a 5-generation family.

**Methods:**

Oral and radiographic examination, linkage analysis, whole genome sequencing, and an immunohistochemical study of Kdf1 during tooth development in the mouse embryo were performed. A protein model was generated.

**Results:**

We report a 5-generation family in which multiple natal teeth, oligodontia, and root maldevelopment manifested with autosomal dominant inheritance. Linkage analysis and whole genome sequencing revealed a novel pathogenic variant c.845T>G; p.Ile282Ser, which cosegregated in 9 affected and 10 unaffected family members. This amino acid Ile282 is highly conserved and is important for the stabilization of a small helical fragment. This stabilization is lost in the Ile282Ser mutant, resulting in disruption of the interaction of KDF1 with its partner proteins, including IKKA, which are important for epidermal proliferation and differentiation and subsequent tooth development.

**Conclusions:**

Our study demonstrates for the first time that natal teeth, tooth agenesis, and root maldevelopment are caused by a *KDF1* variant. Our study highlights the important role of *KDF1* in tooth formation and eruption.

## Introduction

Natal teeth are teeth that are present at birth, whereas neonatal teeth are teeth that erupt within the first 30 days of life.^1^ They are generally of normal primary dentition. However, some may be supernumerary teeth of the primary dentition.^2^, ^3^, ^4^, ^5^ Related complications of natal teeth include feeding issues, traumatic lingual ulceration (Riga-Fede ulceration) and risk of aspiration.^6^ A retrospective study in India showed that all 17 natal or neonatal teeth they examined were radiographically confirmed to be supernumerary teeth.^7^

Natal teeth are three times more common than neonatal teeth. A recent systematic review and meta-analysis of 23 studies in the literature found that 1 in 289 newborns had natal teeth and 1 in 2212 had neonatal teeth.^8^ In one study the incidence of natal and neonatal teeth ranged from 1:2000 to 1:3000.^4^ The prevalence of natal and neonatal teeth appears to depend on population studied. Of the 231 natal teeth from 13 studies reviewed, most of them (95.67%) were mandibular incisors and natal teeth were twice as common in females as in males.^8^ Among 33 infants with 28 natal teeth and 24 neonatal teeth studied in India, all were in the mandibular primary incisor region, and most were in pairs. A positive family history was present in eight cases, suggesting that natal and neonatal teeth may have a hereditary basis.^5^

The prevalence of isolated (nonsyndromic) hypodontia or tooth agenesis ranges from 3% to 10%, while severe tooth agenesis or oligodontia is less common with a prevalence of 0.1% to 0.5%, excluding third molars. Tooth agenesis in primary dentition is rare, with the prevalence ranging from 0.4% to 0.9% in the European population.^9^^,^^10^ There have been infrequent reports of Isolated multiple natal teeth followed by tooth agenesis inherited in an autosomal dominant fashion,^11^, ^12^, ^13^, ^14^ but no gene has been discovered.

Genetic variants in the *Keratinocyte Differentiation Factor 1* (*KDF1*; MIM 616758) gene have been reported in patients with isolated tooth agenesis^15^, ^16^, ^17^, ^18^ and Ectodermal dysplasia 12, hypohidrotic hair-tooth-nail type (ECTD12; MIM 617337).^19^, ^20^, ^21^ KDF1 is known to modulate the optimal balance between cell proliferation and differentiation, which is fundamental and critical for organ formation during development as well as for tissue maintenance postnatally. The *Kdf1* gene is expressed in epidermal progenitor cells from early stages of epidermal development to adulthood. The role of KDF1 is to function upstream of TP63 to regulate the balance between proliferation and differentiation in epidermal progenitor cells^22^ through repression of progenitor cell proliferation by inhibiting TP63, and driving these cells towards terminal differentiation through interaction with STRATIFIN, a cell cycle regulator.^22^ Thus, TP63 is the upstream regulator of STRATIFIN and KDF1. Abnormal Kdf1 leads to uncontrolled cell proliferation on the epidermis, and dysregulation of differentiation, resulting in failure to develop terminal fates.^22^

Natal teeth may be isolated or nonsyndromic, but they may be associated with tooth agenesis.^23^ or part of the genetic syndromes including Ellis-van Creveld syndrome, Pallister-Hall syndrome, and Wiedemann-Rautenstrauch syndrome.^4^ A natal tooth was reported in a Saudi patient who carried a genetic variant (c.753C>A; Phe251Leu) in the *KDF1* gene and was affected by ECTD12.^19^ Three years later, a son of a Saudi mother previously reported to have ECTD12 and the same *KDF1* variant^19^ was born with eight natal teeth^24^ These findings demonstrate a link between the *KDF1* gene and the pathogenesis of natal teeth.

Here we report a novel heterozygous variant (c.845T>G; p.Ile282Ser) in the *KDF1* gene in a 5-generation family affected with a unique dental phenotype including multiple natal teeth, tooth agenesis, and root maldevelopments. The inheritance is autosomal dominant with complete penetrance and variable expressivity over 5 generations in 150 years.

## Materials and methods

### Ethical consideration

Informed consent Informed consent was obtained from the parent of each child. This study involving human participants was approved by the Human Experimentation Committee of the Faculty of Dentistry, Chiang Mai University (no. 25/2020) and by Cedars-Sinai Medical Center IRB Birth Defects Research Study ID:0463 and was performed in accordance with the ethical standards of the 1964 Declaration of Helsinki and its later amendments or comparable ethical standards.

### Patient recruitment

**Fig. 1:**
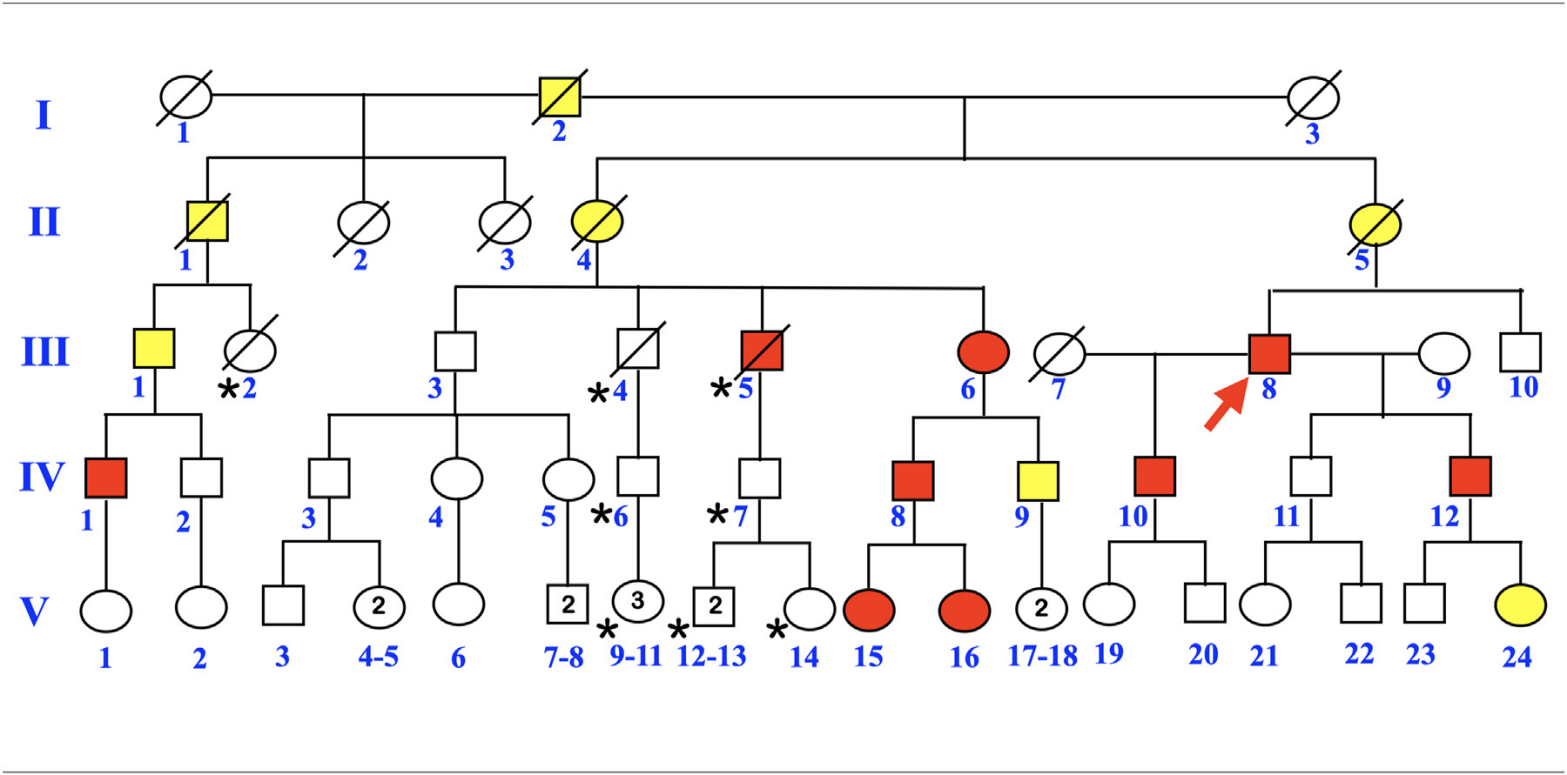
Pedigree of a 5-generation Caucasian family affected with multiple natal teeth, tooth agenesis, and root maldevelopments. Nine affected patients had tooth agenesis with natal teeth (III-5, III-6, III-7, IV-1, IV-8, IV-10, IV-12, V-15, and V-16) and 2 patients had tooth agenesis with no apparent natal teeth (IV-9 and V-24), while the presence or absence of natal teeth could not be confirmed in 5 older individuals with tooth agenesis (I-2 II-1, II-4, II-5, III-1). (Red depicts tooth agenesis and natal teeth; yellow depicts tooth agenesis; *depicts those who were not studied.)

**Fig. 2:**
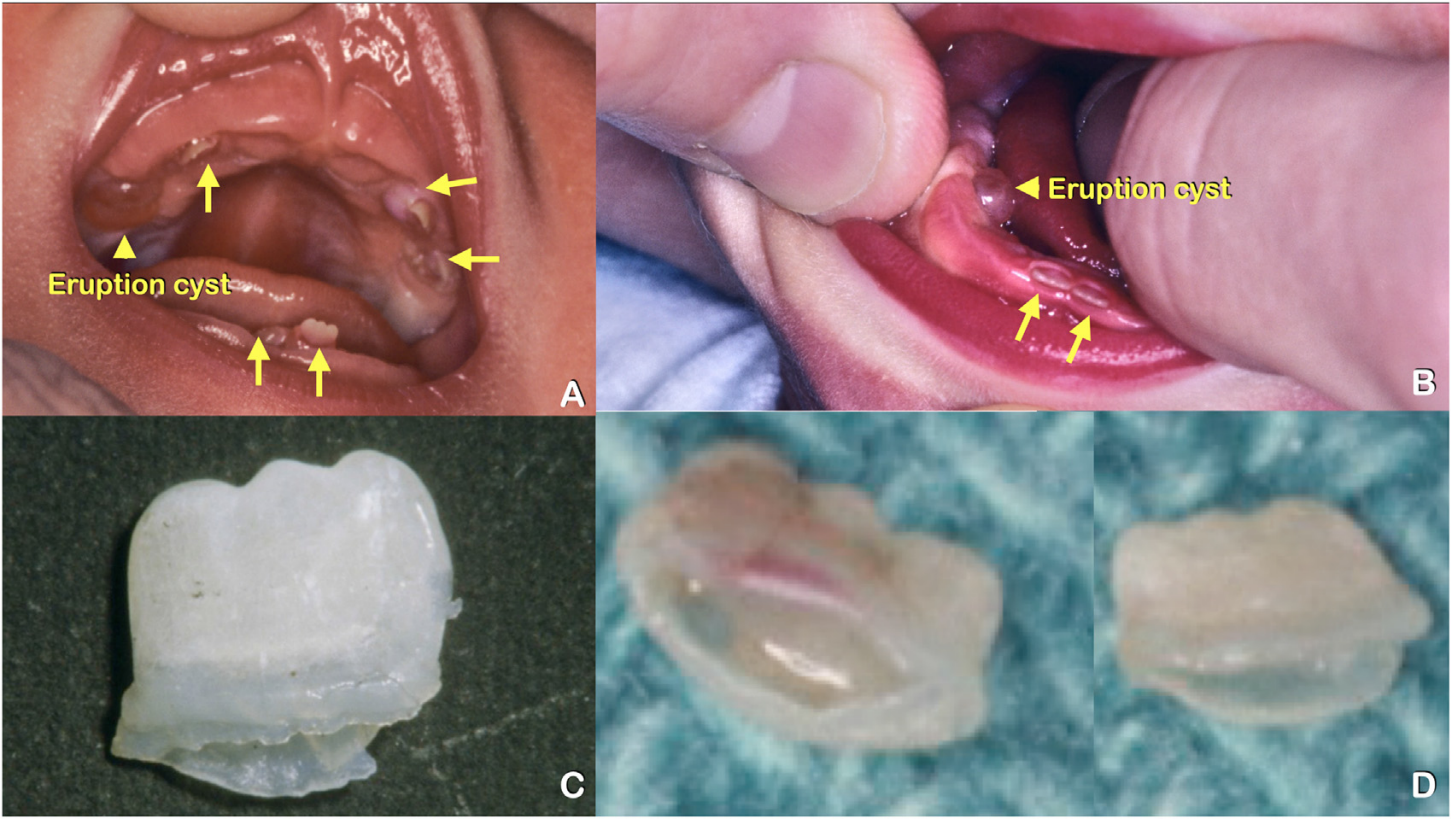
Patient IV-10. (A and B) Multiple natal teeth (arrows), and eruption cysts (arrowheads). (C and D) Extracted natal teeth. Note the thin tooth structure and shortened roots.

Nine affected (III-8, IV-1, IV-8, IV-9, IV-10, IV-12, V-15, V16, V-24) and 10 unaffected (III-3, III-10, IV-2, IV-11, V-1, V-17, V-18, V-19, V-20, V-23) members of this family were recruited into this study (Figure 1). Most of the information was gathered by the proband (J.G.) who is a board-certified medical geneticist. The information on natal teeth was obtained by asking the individuals or their parents. Panoramic radiographs were obtained from 8 affected family members and 1 unaffected family member. The number of natal teeth of most family members was 2 to 3 teeth. Individual IV-10 had seven natal teeth of which two of them were covered by eruption cysts (Figure 2A, B). The extracted mandibular incisors were shortened, thin, and rootless (Figure 2C, D).

Four ml of venous blood was collected into EDTA-containing blood collection tubes (Vacutainer, Becton, Dickinson). The genomic DNA of all patients was isolated from blood using a standard protocol.

### Linkage analysis

Linkage analysis was performed on 10 affected and 18 unaffected family members (Supplementary Materials).

### Whole genome sequencing

Quad whole genome sequencing was performed on III-8, III-9, IV-11 and IV-12 (Supplementary Materials).

### Protein model

AlphaFold models of the KDF1-IKKA complex were produced using our in-house implementation of AlphaFold_multimer^25^ and the AlphaFold3 online server.^26^ The analysis was carried out as previously described.^27^ Structures were visualized with PyMOL (pymol.org).

### Immunohistochemical study of Kdf1 during tooth development in mouse embryo

Heads were dissected from CD1 mice, fixed with 4% paraformaldehyde, wax embedded, and serially sectioned at 7 µm. Decalcification using 0.5 M EDTA (pH 7.6) was performed after fixation for E18.5, P10, and P15. Sections were incubated at 4°C overnight with the antibody to Kdf1 (Abcam). The tyramide signal amplification system (Parkin Elmer Life Science) was used for detecting the primary antibody.

## Results

### Linkage analysis, whole genome sequencing, and bioinformatic analysis

**Fig. 3:**
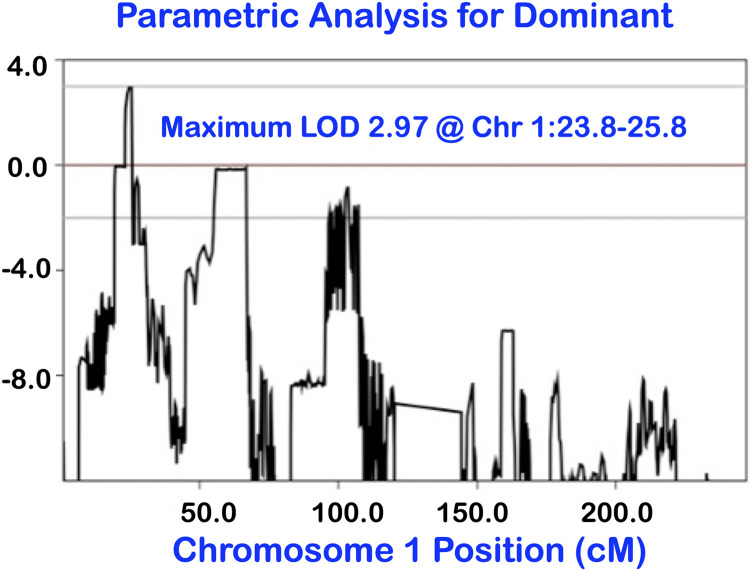
Linkage peaked at 1p36.11. This dental phenotype is mapped to a 2 Mb region on chromosome 1p36.11 between *LOC284632* and *GRHL3* with an LOD score of 2.97.

In 2010, this unique dental phenotype was mapped to a 2 Mb region on chromosome 1p36.11 between *LOC284632* and *GRHL3* with LOD score 2.97 (parenTDT, *P* = .005 for rs11249039, rs11249045, or rs7526505) (Figure 3). 311 SNPs located on chromosome 1, 23.6 to 25.8 Mb were modelled jointly for both linkage and association (LAMP). The peak association LOD = 1.8 @ rs11249045 & rs7526505, *P* = .0035 was located between *LOC284632* and *GRHL3*).^28^

**Fig. 4:**
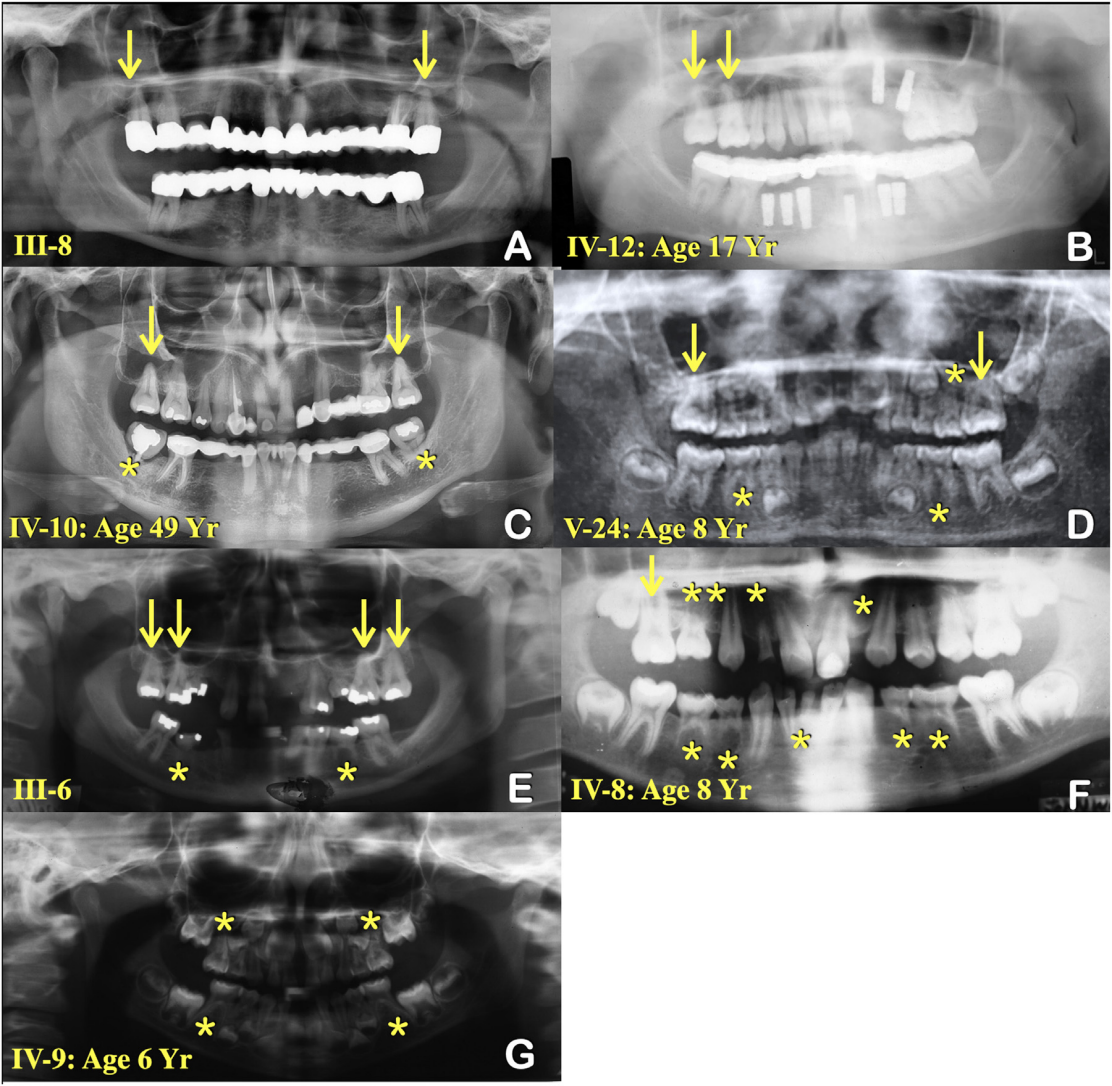
Panoramic radiographs of patients with the *KDF1* variant. (A) Patient III-8. Prosthetic bridges in the areas of tooth agenesis. Unseparated roots of the permanent molars (arrows). (B) Patient IV-12. Dental implants in the areas of tooth agenesis. Unseparated roots of the permanent molars (arrows). (C) Patient IV-10. Prosthetic bridges in the areas of tooth agenesis. Unseparated roots of the permanent molars (arrows). Rootless teeth (asterisks). (D) Patient V-24. Tooth agenesis (asterisks). Taurodontism (arrows). (E) Patient III-6. Tooth agenesis (arrows). Unseparated roots of the permanent molars (arrows). (F) Patient IV-8. Tooth agenesis (asterisks). Taurodontism (arrows). (G) Patient IV-9. Tooth agenesis (asterisks).

Whole genome sequencing revealed a novel pathogenic c.845T>G; p.Ile282Ser variant in the *KDF1* gene on 1p36.11 where the linkage had previously been established. Segregation analysis in 9 affected (III-8, IV-1, IV-8, IV-9, IV-10, IV-12, V-15, V16, V-24) and 10 unaffected (III-3, III-10, IV-2, IV-11, V-1, V-17, V-18, V-19, V-20, V-23) members of this family confirmed that this *KDF1* variant segregated with the dental phenotype in otherwise normal family members with no ectodermal dysplasia or other recognizable syndrome (Figures 1, 2, 4).

According to gnomAD, this variant is absent from control populations and has not been reported in individuals. Therefore, it is considered to be novel. The p.Ile282Ser variant has a CADD score of 28 and is predicted to be probably damaging (0.989) and disease-causing (prob:0.999997735000506) by PolyPhen-2 and MutationTaster, respectively.

### Immunohistochemical study of Kdf1 expression in mouse embryos

**Fig. 5:**
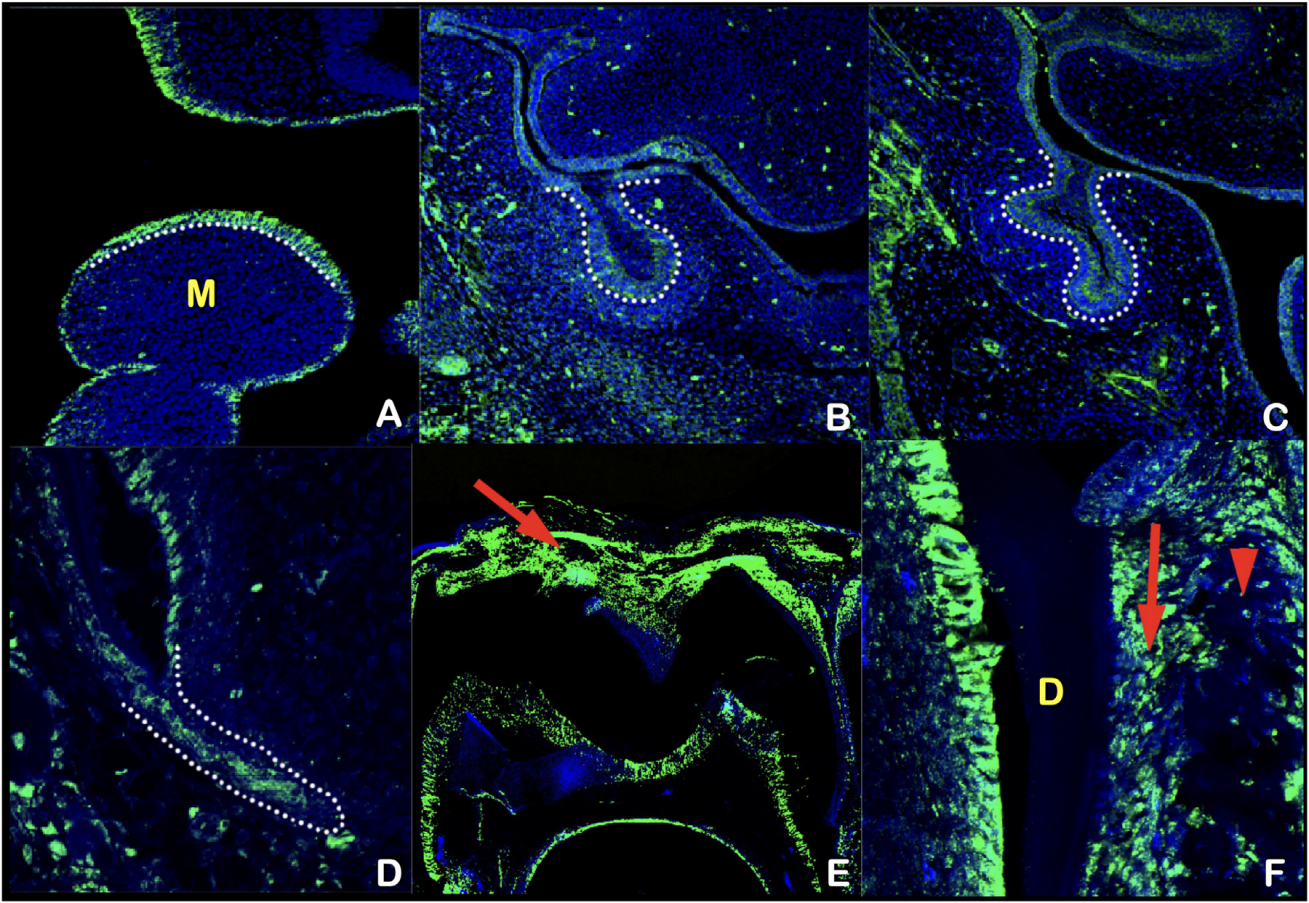
**Kdf1 expression in tooth development.** Frontal sections showing immunohistochemistry of Kdf1 in mouse tooth germs at embryonic day (E) 9.5 (A), E13.5 (B), E14.5 (C), and postnatal day (P) 10 (D) and P15 (E and F). The tooth epithelium was outlined by white dots. M, the mandibular process. Arrow indicating tissue above the tooth germ (E). Arrow and arrowhead indicating periodontal ligament and alveolar bone, respectively (F). D, dentin.

Tooth agenesis was also observed in some patients, and Kdf1 was expressed in the epithelium of mandibular processes before the initiation of tooth development and in the dental epithelium at the bud and cap stage (Figure 5A-C), which is consistent with a previous report by Zeng et al.^15^ Some patients had root maldevelopments, and Kdf1 was expressed in Hertwig epithelial root sheath (Figure 5D). Natal teeth or premature tooth eruption was also observed in some patients. Kdf1 was expressed in the tissue surrounding the tooth germ just before eruption (Figure 5E, F).

### Protein model

**Fig. 6:**
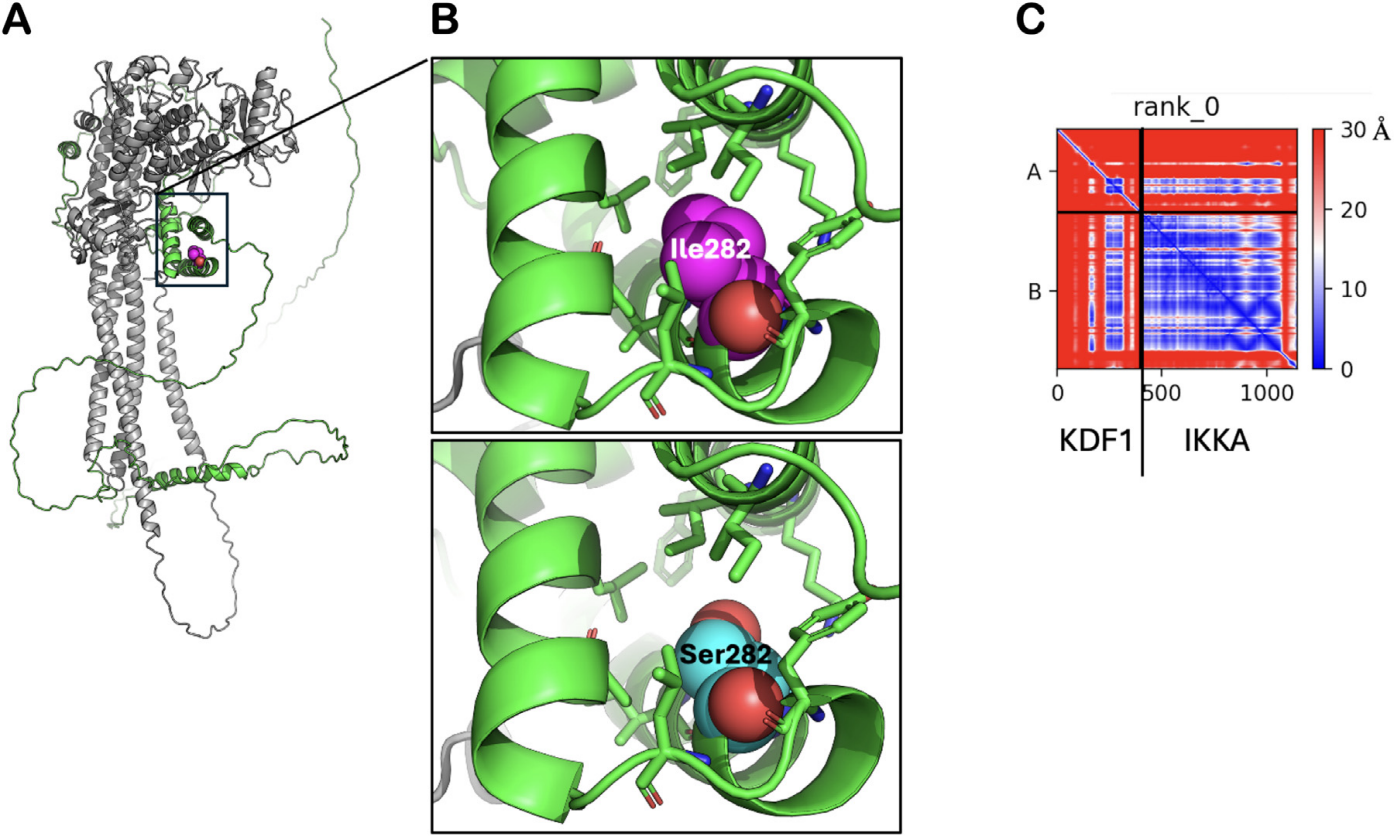
(A) Model of the interaction between KDF1 (green) and IKKA (grey). Ile 282 is shown as a magenta sphere model. (B) Zoom into the boxed region of (A). Ile is shown as a magnetosphere model. Side chains of residues of KDF1 establishing hydrophobic residues with Ile282 are shown as stick models. (C). Predicted aligned error (PDE) matrix, colour-coded from blue (0 Å) to red (30 Å).

KDF1 is predicted by AlphaFold to be a mostly disordered protein without stable tertiary structure (alphafold.ebi.ac.uk/entry/Q8NAX2). However, already in the monomeric structure, Ile282 is positioned in the only region where two helices are predicted to form a stable association, as illustrated by a low predicted alignment error of this region (residues 265-305) (Figure 6A-C). The sequence change, p.Ile282Ser, which was found in our patients replaces isoleucine, which is neutral and nonpolar, with serine, which is neutral and polar, at codon 282 of the KDF1 protein. Ile282 has a critical role in stabilizing this connection between the helices through hydrophobic interactions. This stabilization would be lost in the Ile282Ser mutant. In line with published experimental evidence, AlphaFold predicts a strong association of KDF1 with IKKA. This association is mediated by a structural helical element formed around Ile282. The mutation of the Ile282 into a smaller and hydrophilic serine would critically affect this helical structure, and hence its interaction with IKKA and possibly other partners (Figure 6A-C).

## Discussion

We report a 5-generation family affected with multiple natal teeth, tooth agenesis, and root maldevelopments. Seven affected patients had tooth agenesis and natal teeth (III-8, IV-1, IV-8, IV-10, IV-12, V-15, V16) but 2 patients (IV-9 and V-24) did not have natal teeth evident at birth. We could not determine whether or not the 5 senior members of this family (I-2, II-1, II-4, II-5, and III-1) with tooth agenesis had natal teeth because they had passed away or did not recall. Since the birth of the first generation (individual I-2) in 1874, this unique dental phenotype has been passed on in an autosomal dominant manner through 5 generations for 150 years. There were some difficulties in getting records or memories regarding the presence and location of natal teeth. Since we are not sure if some of them (I-2, II-1, II-4, II-5, and III-1) had natal teeth, we assigned them as having only tooth agenesis. Regarding the number of natal teeth at birth, unfortunately, for some individuals, there was no record or memory of the number of teeth at birth by their parents.

### Pathogenicity of p.Ile282Ser variant

The amino acid Ile282 is highly conserved. It is present in 411 species of 421 jawed vertebrates. It is interesting that 10 species of frogs and toads have the amino acid Leu282 at this position. The amino acid Ile282 plays a critical role in stabilizing this connection between the helices through hydrophobic interactions. This stabilization is lost in the Ile282Ser variant. KDF1 has a strong association with IKKA. The change of Ile282 to a smaller and hydrophilic serine as a result of a mutation would critically affect this helical structure and thus disrupt its interaction with IKKA and possibly other partners.

### KDF1 and isolated tooth agenesis

The family we report herein with a pathogenic variant (c.845T>G; p.Ile282Ser) in the *KDF1* gene had isolated tooth agenesis, which in most cases was preceded by multiple natal teeth. However, we do not know if the positions of the natal teeth are related to the positions of the missing teeth. Most affected adults were missing at least 14 to 17 teeth, most commonly lateral incisors, canines, and first and second premolars, although one affected adult (IV-9) was only missing 4-second premolars and did not have any apparent natal teeth. The association of tooth agenesis and a *KDF1* variant in this family is supported by previous reports.^15^, ^16^, ^17^, ^18^ and Kdf1 is expressed in the dental epithelium of the mandibular processes prior to the onset of tooth development and at the bud and cap stages. Notably, the association of natal teeth and tooth agenesis is extremely rare but has been reported.^23^ This association is useful for genetic counselling as patients with natal teeth may have tooth agenesis in the future.

**Fig. 7:**
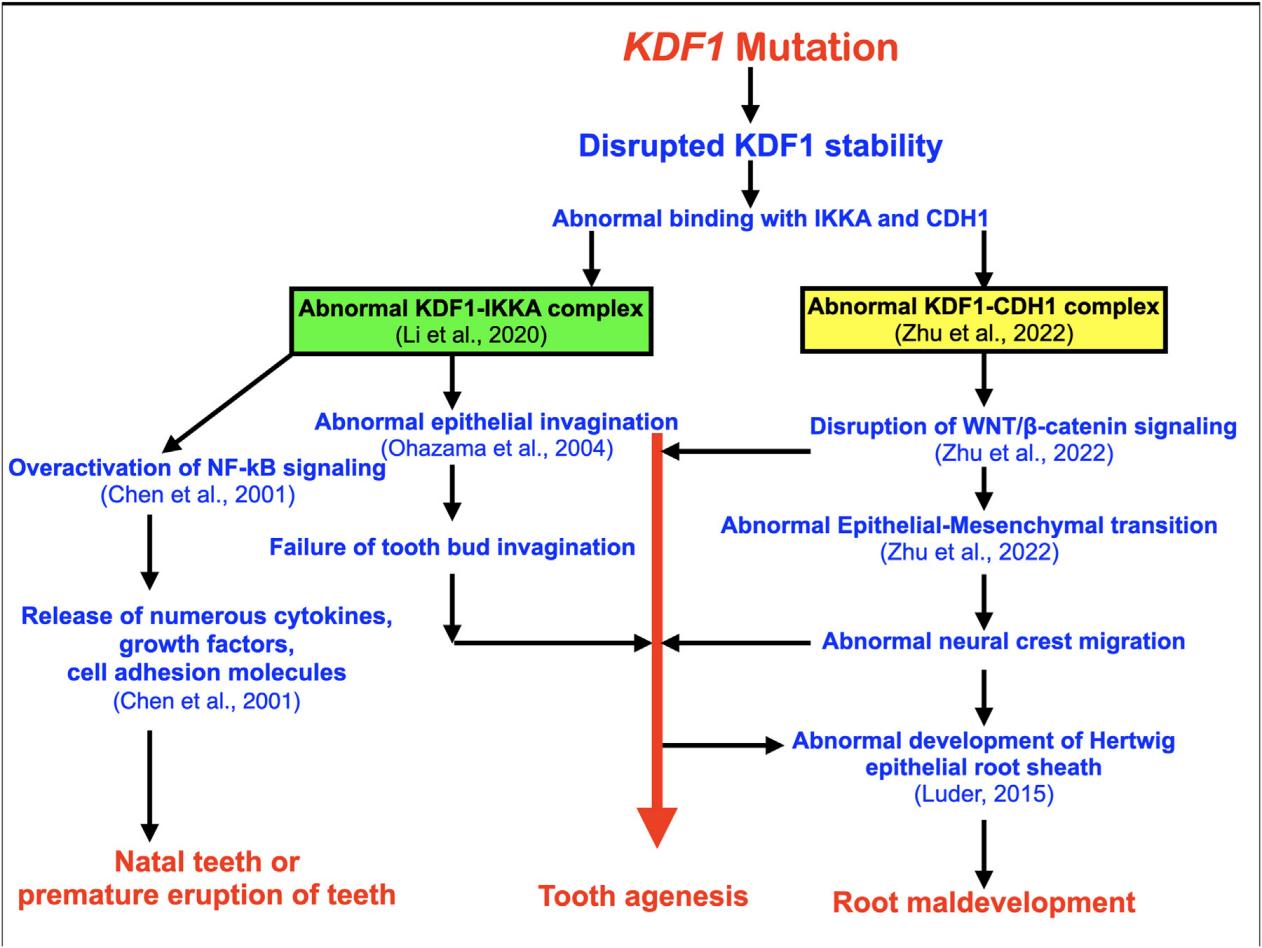
Flowchart of hypothetic pathogenesis of natal teeth, tooth agenesis, and root development as a result of a genetic variant in the *KDF1* gene. A genetic variant in the *KDF1* gene leads to abnormal binding of KDF1 and IKKA and KDF1 and CDH1, abnormal KDF1-IKKA and KDF1-CDH1 complexes, overactivation of NF-kB signalling, disruptive WNT/B-catenin signalling, abnormal epithelial invagination, and subsequent isolated dental anomalies.

The interaction between KDF1 and IKKA is essential for epidermal differentiation^22^^,^^29^ and it mediates the regulation of deubiquitination in epidermal differentiation.^29^^,^^30^ IKKA is important for tooth and skin development. Ablation of either *Kdf1* or *Ikkα* in mice leads to similar abnormalities in skin development, particularly in skin epidermal differentiation.^29^ According to the protein models, the Ile282Ser variant would be expected to disrupt this interaction and subsequent tooth development. Ikka plays an important role in cusp development via the NF-kB signalling pathway. It regulates the direction of epithelial invagination into the underlying mesenchyme of hair and teeth, through NF-kB signalling (Figure 7).^31^

Notably, KDF1 also interacts with CADHERIN 1 (E-cadherin) (CDH1; MIM 192090) to form the KDF1-CADHERIN 1 complex, which plays an important role in activating WNT/β-catenin signalling to regulate epithelial-mesenchymal transition,^30^ the process critical for neural crest cells to migrate and participate in the development of the craniofacial structures including teeth.^30^^,^^32^ This is supported by the presence of tooth agenesis in a number of patients with *CDH1* mutations.^33^, ^34^, ^35^ Therefore, disruption of WNT/β-catenin signalling and epithelial-mesenchymal transition secondary to impaired interactions between KDF1 and IKKA and KDF1 and CDH1 as a result of a *KDF1* variant may contribute to dental anomalies (Figure 7).

### KDF1 variant and natal teeth

Most natal teeth found in this family were immature as they appeared hypoplastic and with no roots. The finding of enamel defects in *Kdf1* knock-in mice supports this finding in our patients.^36^ Natal teeth have been previously reported in patients with a *KDF1* variant, suggesting the link between *KDF1* and natal teeth.^19^^,^^24^ The patient reported by Aljohar et al^24^ was born with eight natal teeth. The question is how alterations in KDF1 result in premature eruption of primary teeth.

Tooth eruption is a complex biological process that regulates the precise gradual movement of tooth buds from their initial positions in the jawbone to their functional positions in the oral cavity.^37^, ^38^, ^39^ Tooth-eruption requires both the formation of an eruption pathway and the generation of motive forces that originate from the dental follicle, osteogenic activities in the alveolar bone, the development of the periodontal ligament, and traction forces from associated fibres. The dental follicle is indispensable in the tooth-eruption process. It has been shown that a metal object replacing a tooth in a dental follicle still erupted, indicating that the eruption of a tooth does not even require the tooth, but rather a functional dental follicle.^38^ It seems that the premature eruption of teeth or natal teeth results from malfunction of the ‘delivery system’, not of the package (teeth). The dental follicle plays a critical role in the process of tooth eruption by regulating bone remodelling through the activities of osteoblasts and osteoclasts. It is hypothesized that alteration in KDF1 because of this *KDF1* variant may affect the dental follicle cells resulting in the ‘speedy delivery’ of natal teeth.

Most of the previously reported natal teeth are the mandibular central incisors.^8^ However, natal teeth of the posterior teeth have been reported but are very rare.^24^^,^^40^, ^41^, ^42^, ^43^, ^44^ Multiple (more than 2) natal teeth are extremely rare.^14^^,^^45^, ^46^, ^47^ The presence of natal or neonatal teeth in the same families^1^^,^^5^^,^^11^^,^^48^^,^^49^ or in twins^50^, ^51^, ^52^, ^53^, ^54^ supports their hereditary basis.

Natal teeth are usually present with inflammatory tissue around them. Patient IV-10 was born with eruption cysts and inflammatory tissue around the natal teeth. This suggests a relationship between premature tooth eruption and inflammation of the surrounding tissue. Our immunohistochemical study showed that Kdf1 was expressed in the tissue surrounding the tooth germ just before eruption. It is hypothesized that a genetic variant in the *KDF1* gene could disrupt its interaction with IKKA, lead to overactivation of NF-kB signalling, and result in inflammation of surrounding tissues and premature eruption of primary teeth. In addition to inflammation of surrounding tissues, overactivation of NF-kB signalling would cause cells especially lymphocytes to release numerous cytokines, growth factors, and cell adhesion molecules that could accelerate the eruption of teeth (Figure 7).^55^

### KDF1 variant and root maldevelopments

Root maldevelopments found in our patients include taurodontism, unseparated roots of permanent molars, and rootless teeth. Notably, taurodontism and single-rooted primary molars have been reported in a patient with a *KDF1* variant.^16^^,^^17^ The findings of root maldevelopments support the role of the *KDF1* gene in root morphogenesis. Root formation is regulated by sequential and reciprocal interactions between the Hertwig epithelial root sheath, which is derived from the cervical loop of the enamel organ and the surrounding mesenchyme. Hertwig epithelial root sheath determines the number, shape, and length of roots. Our immunohistochemical study showed that Kdf1 is highly expressed in the Hertwig epithelial root sheath during early root development, supporting the important role of the *KDF1* gene in root morphogenesis. Alteration in KDF1 is likely to disrupt its interaction with IKKA and impair epithelial invagination.^56^ Proper root development requires invagination of epithelial diaphragm.^57^, ^58^, ^59^ It is hypothesized that the *KDF1* variant disrupts the invagination of the Hertwig epithelial root sheath and epithelial diaphragm, resulting in root maldevelopment in our patients (Figure 7).

We are convinced that the *KDF* variant c.845T>G; p.Ile282Ser is associated with the isolated dental anomalies found in this family because this variant is novel and segregates in nine affected and 10 unaffected family members, leaving a negligible probability that this complete segregation is by chance. In addition, the *KDF1* gene is located at 1p36.11, where the linkage was mapped. This variant has a CADD score of 28 and is predicted by PolyPhen-2 and MutationTaster to be probably damaging and disease-causing, respectively. Whole genome sequencing of the patients did not reveal any other rare genetic variants in other known tooth-related genes.^60^^,^^61^ Finally, previous reports of *KDF1* variants in patients with natal teeth, tooth agenesis, and root maldevelopment support our hypothesis (Figure 7). Clinicians should be aware that patients with natal teeth may have multiple tooth agenesis in the future. If they have tooth agenesis that is radiographically evident at a very young age, patients and their parents should be informed that the patients may require orthodontic treatment and dental implants later in life.

## Conclusion

Our study demonstrates for the first time that the newly recognized dental syndrome including natal teeth, tooth agenesis, and root maldevelopment is caused by a *KDF1* variant. It is hypothesized that alteration of KDF1 as a result of the *KDF* variant disrupted the interactions between KDF1 and IKKA and CDH1 and resulted in aberrant WNT/β-catenin signalling, impairing the balance between cell proliferation and differentiation, aberrant epithelial-mesenchymal transitions, and subsequent isolated dental anomalies.

## Author contributions

John M. Graham Jr, Pedro A. Sanchez-Lara, Atsushi Ohazama, Katsushig Kawasaki, Stefan T. Arold: conceptualization, investigation, methodology, analysis, and writing/reviewing/editing; Piranit Nik Kantaputra: conceptualization, investigation, methodology, analysis, project supervision, grant management, writing/reviewing/editing.

## Funding

This research was supported by Chiang Mai University (P.K.) and The Genomics Thailand Research Grant of the Health System Research Institute of Thailand (64-123) (P.K.). The research by STA was supported by funding from King Abdullah University of Science and Technology (KAUST) – KAUST Center of Excellence for Smart Health (KCSH), under award number 5932. For computer time, this research used the resources of the KAUST Supercomputing Laboratory at KAUST (S.T.A.).

## Conflict of interest

The authors declare that they have no known competing financial interests or personal relationships that could have appeared to influence the work reported in this article.

## Appendix Supplementary materials

Image, application 1

## Footnotes

Supplementary material associated with this article can be found in the online version at doi:10.1016/j.identj.2025.100860.
