# Supplementary material for: A Novel KDF1 Variant is Associated With Multiple Natal Teeth, Tooth Agenesis, and Root Maldevelopment
Source: Int Dent J. 2025 Jun 23;75(4):100860. doi: 10.1016/j.identj.2025.100860 (PMC12240073; doi:10.1016/j.identj.2025.100860)
Supplement: Supplementary file 1 [file mmc1.docx]

**Supplementary materials**

**Linkage Analysis**

In 2010 affected and unaffected members of the were recruited to perform a linkage analysis to identify the locus for this unique phenotype of multiple natal teeth, tooth agenesis, and root maldevelopment. DNA samples from were isolated from the blood of 28 family members (10 affected: III-1, III-5, III-6, III-8, IV-1, IV-8, IV-9, IV-10, IV-12, V-15) and 18 unaffected (III-7, III-9, III-10, IV-2, IV-3, IV-4, IV-5, IV-6, IV-7, IV-11, and V-19) including seven unaffected spouses not shown on the pedigree. One subject was misclassified (IV-9) because he was not born with natal teeth and was less severely affected with selective tooth agenesis, but there were enough participants to establish linkage to 1p36.1. Some family members had passed away or chose not to participate in the segregation analysis 13 years later.

For linkage analysis, the participants were genotyped on an Illumina OMNI-express chip using 733,120 SNPs with 361,754 311SNPs for analyses. The data set was reduced to 21,862 SNP clusters with r2<0.1 by LD-based SNP pruning (plink). The pedigree was divided into three branches to make computation possible. Parametric linkage analysis was performed using dominant model (MERLIN)^1^ and mapped to an approximately 2Mb segment on chromosome 1p36.11 with LOD score 2,97 at 23.8 Mb to 25.8 MB (Merlin) between *LOC284632* and *GRHL3. KDF1* was not known to be a selective tooth agenesis gene at this time.

**Whole genome sequencing**

Quad whole genome sequencing was performed on III-8, III-9, IV-11 and IV-12 (Supplementary materials). Whole genome short read sequencing was performed using the Illumina® DNA PCR-Free Prep, Tagmentation, followed by next generation sequencing. Analyses were performed to detect, analyze and report clinically relevant variants using the Variantyx Genomic Intelligence® platform version 3.9.0.0. No other variants in genes associated with tooth agenesis or candiate genes were identified.

**REFERENCES**

1. Abecasis GR, Cherny SS, Cookson WO, Cardon LR. Merlin--rapid analysis of dense genetic maps using sparse gene flow trees. *Nat Genet*. Jan 2002;30(1):97-101. doi:10.1038/ng786
